# Estradiol effects on the dopamine transporter – protein levels, subcellular location, and function

**DOI:** 10.1186/1750-2187-1-5

**Published:** 2006-12-05

**Authors:** Cheryl S Watson, Rebecca A Alyea, Bridget E Hawkins, Mary L Thomas, Kathryn A Cunningham, Adrian A Jakubas

**Affiliations:** 1 Department of Biochemistry & Molecular Biology, Univ. of Texas Medical Branch, Galveston TX 77555-0645, USA; 2 Department of Pharmacology & Toxicology, Univ. of Texas Medical Branch, Galveston TX 77555-1031, USA

## Abstract

**Background:**

The effects of estrogens on dopamine (DA) transport may have important implications for the increased incidence of neurological disorders in women during life stages when hormonal fluctuations are prevalent, e.g. during menarche, reproductive cycling, pregnancy, and peri-menopause.

**Results:**

The activity of the DA transporter (DAT) was measured by the specific uptake of ^3^H-DA. We found that low concentrations (10^-14 ^to 10^-8 ^M) of 17β-estradiol (E_2_) inhibit uptake via the DAT in PC12 cells over 30 minutes, with significant inhibition taking place due to E_2 _exposure during only the last five minutes of the uptake period. Such rapid action suggests a non-genomic, membrane-initiated estrogenic response mechanism. DAT and estrogen receptor-α (ERα) were elevated in cell extracts by a 20 ng/ml 2 day NGFβ treatment, while ERβ was not. DAT, ERα and ERβ were also detectable on the plasma membrane of unpermeabilized cells by immunocytochemical staining and by a fixed cell, quantitative antibody (Ab)-based plate assay. In addition, PC12 cells contained RNA coding for the alternative membrane ER GPR30; therefore, all 3 ER subtypes are candidates for mediating the rapid nongenomic actions of E_2_. At cell densities above 15,000 cells per well, the E_2_-induced inhibition of transport was reversed. Uptake activity oscillated with time after a 10 nM E_2 _treatment; in a slower room temperature assay, inhibition peaked at 9 min, while uptake activity increased at 3 and 20–30 min. Using an Ab recognizing the second extracellular loop of DAT (accessible only on the outside of unpermeabilized cells), our immunoassay measured membrane vs. intracellular/nonvesicular DAT; both were found to decline over a 5–60 min E_2 _treatment, though immunoblot analyses demonstrated no total cellular loss of protein.

**Conclusion:**

Our results suggest that physiological levels of E_2 _may act to sequester DAT in intracellular compartments where the transporter's second extramembrane loop is inaccessible (inside vesicles) and that rapid estrogenic actions on this differentiated neuronal cell type may be regulated via membrane ERs of several types.

## Background

Dopamine (DA) is a catecholamine neurotransmitter important in myriad brain functions. Disruptions of DA neurotransmission are associated with a wide range of pathological conditions. Gender differences in the expression of some of these diseases [1], as well as fluctuations in estrogen levels over the life span in women [2,3], suggest the possibility that estrogens may play a role in modulating DA signaling [4,5]. In females the predominant estrogen, estradiol (E_2_), typically rises from prepubertal amounts of ~20 pM, to as high as a 2–3 nM peak cycle concentration in adults, fluctuates during peri-menopause, and eventually falls to chronically lower postmenopause levels. In pregnancy E_2 _levels can rise as high as 20 nM, declining precipitously after parturition. In addition, other estrogens (estriol, estrone) also change. Estrogens that fluctuate dramatically and then decline at menopause can be correlated with the onset of some mood disorders [6]. Pubertal fluctuations in estrogen levels are associated with mood variations in young girls [7]. Some women experience mood disturbances as a function of monthly cyclic hormonal fluctuations (premenstrual syndrome or premenstrual disphoric disorder, or in extreme cases, premenstrual dementia [8-10]). Increased body fat actually protects against cycle-based mood swings [11] and surgical menopause-based depression, probably by serving as a depot for lipophillic hormones (including estrogens) that buffer large changes [12]. Therefore, in patients where these changes are excessive, disturbances of behavior may result; it is important to understand the cellular mechanisms through which estrogens operate across this wide range of physiological levels [13].

There are other specific gender-biased cognitive or neural function-based medical conditions that can involve DA synaptic signaling. Crises in schizophrenia/bi-polar disorders can sometimes be directly correlated to menstrual cycle hormonal fluctuations [14]. There is a sharp rise in the incidence of Alzhiemer's disease after menopause [15]. Some diseases that involve DA neurotransmission are less prevalent or different in premenopausal females vs. postmenopausal females and males (Parkinson's, Tourette's, ADHD [16-20]), also suggesting an influence of estrogens on disease status. Other studies suggest an involvement of estrogens in cognitive function [21] and attention [22-24]. Females are also more vulnerable to cocaine use than are males [25-27]. Thus estrogens probably broadly influence the status of neural signal transmission.

While estrogens acting through their intracellular receptors are known to regulate gene transcription, it is becoming increasingly clear that estrogens can also initiate cellular effects at the membrane [reviewed in [28,29]]. Unlike transcriptional events, membrane-initiated events have the capacity to be dynamically regulated over short time frames and are not necessarily dependent on protein synthesis or degradation. Furthermore, membrane-initiated events can best be rapidly detected in cell assay systems in which estrogens can be experimentally controlled rapidly. Thus, these nongenomic actions are frequently referred to as "rapid" actions of estrogens. However, sustained short-term effects of estrogens, or downstream mechanisms they initiate, can lead to more long-term consequences in animals.

The primary mechanism through which DA levels are regulated in the synapse is via reuptake by the DA transporter (DAT), which is one target of action of a variety of neuroactive drugs including antidepressant agents [30]. Thus, the work reported here was undertaken to characterize a cell culture system that expresses both DAT and membrane-associated estrogen receptors (ERs) that could be utilized to investigate the hypothesis that estrogen regulates DAT activity via rapid, nongenomic mechanisms. We chose a well-known model for neuronal cellular responses involving the regulation of neurotransmitter transporters and receptors, the PC12 pheochromocytoma cell line, in which the presence of intracellular estrogen receptors had previously been reported [31-33]. We examined the ERs that are possibly involved (ERα [34], ERβ [35,36], and GPR30 [37-39]) and ER-mediated functional responses that may explain estrogenic effects on neurotransmitter regulation in the synapse. Specifically, we focus on rapid regulation of the DAT leading to changes in synaptic DA levels likely to be involved in DA-mediated behavioral responses. Understanding a mechanistic role for estrogens in modulating this transporter should suggest new therapeutic targets and regimens tailored for female vs. male patients in the treatment of behavioral disturbances exacerbated by fluctuating estrogens.

## Results

### DAT is upregulated by 2 days of NGF treatment in the PC12 pheochromocytoma cell model

**Figure 1:**
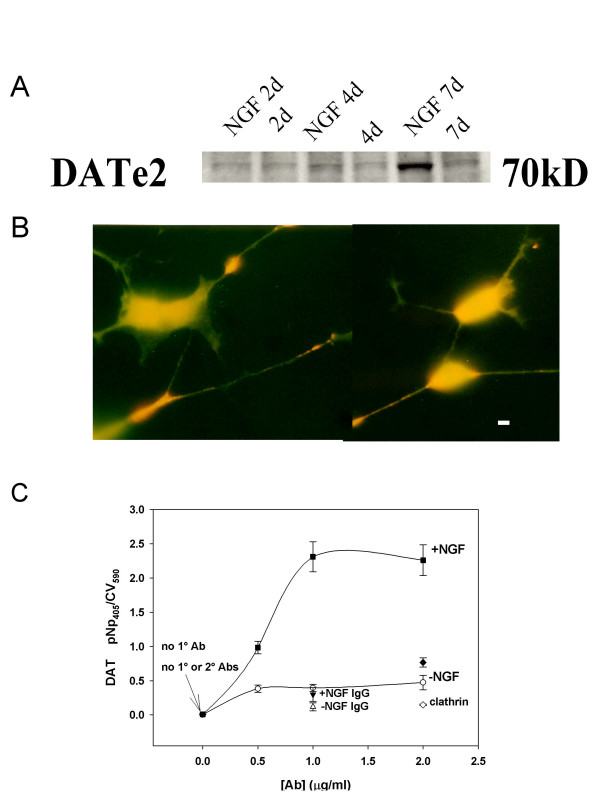
**DAT proteins are present in the membranes of PC12 cells and are elevated by 2–7 day NGFβ treatment**. **A. **Cell lysate immunoblots with Ab to the second extracellular loop of DAT, showing an increase in DAT protein levels due to a 20 ng/ml NGFβ treatment over a time course of 2 (NGF 2d), 4 (NGF 4d) and 7 (NGF 7d) days, compared to controls without NGFβ treatment (2d, 4d, and 7d). Representative of 2 experiments. **B. **Staining of nonpermeablized fixed cells with the same DAT e2 Ab. Fluorescent images viewed with an FITC filter were photographed. Vector Red appears as orange-red signal on a background of yellow-green autofluorescence (typically seen with this cell fixation protocol). Note the staining of cell bodies, especially at the growth cones (overexposed), and the smaller amount of staining on processes of these NGFβ-differentiated cells. The bar represents 2 μm. **C. **DAT levels are stimulated ~8-fold on day 2 of NGFβ treatment, as demonstrated by the plate immunoassay of fixed cells using the same e2 DAT-specific Ab; 2° Ab conjugated to alkaline phosphatase was used to generate paranitrophenol (pNp) colorimetric signals, which were normalized to the cell number determined in each well by the crystal violet (CV) assay. Symbols are +NGF (■); -NGF (○). Controls: NGF-treated cells probed with a nonspecific IgG Ab is labeled as "+NGF IgG" (▼) "-NGF IgG" IgG (△); clathrin (◇); no primary Ab (no 1° Ab) and no primary or secondary Ab (no 1°, no 2° Abs) are at the origin. A very low clathrin Ab signal under these nonpermeabilizing conditions demonstrated the lack of inadvertent permeabilization of the cells in these assays. This graph represents the average values from 3 experiments ± S.E.M.

Previous studies on NGF treatments of PC12 cells focused on an enhanced differentiated cell morphological profile as an endpoint that required relatively long treatment periods (upregulation of DAT after two weeks of treatment with 50 ng NGF [40]). We sought to achieve functional enhanced DAT levels in a shorter time frame. To develop a cell model in which robust DAT responses could be monitored for estrogen regulation after shorter differentiation times, we chose to assay DAT levels directly via an immunofluorescence assay. We treated PC12 cells with low concentrations of NGFβ for 2, 4, and 7 days. An immunoblot of DAT protein from NGF-treated cells detected an increase in DAT protein by day 4 of NGF treatment (Fig. 1A). Fig. 1B shows the appearance of DAT on the membrane of 2 day NGF-treated cells, where DAT is evident on both cell bodies and processes. Next we adapted our immunocytochemistry conditions to a quantitative immunoassay for nonpermeabilized fixed-cells in 96-well plates, similar to a previous assay we developed for other extracellular and intracellular antigens [35,41]. This assay (shown in Fig. 1C) assessed only membrane DAT (under these nonpermeabilizing conditions) by recognizing the second extracellular loop of the DAT protein [42]. We optimized fixation conditions to prevent antibody (Ab) from entering the cells (as shown by the low value for the clathrin Ab control). Clathrin, lying just under the membrane surface, is an abundant antigen, and gives a large signal in this assay when cells are permeabilized by adding detergent during the fixation process (not shown). The NGF-treated cells show an ~8-fold increase in DAT levels vs. untreated cells after just 2 days. These data show a concentration curve with increasing Ab to determine the single concentration of Ab that would saturate the antigen for future one-point assays (1 μg/ml). Both untreated and NGF-treated levels can be measured by this assay (both are above the background values obtained using no 1° Ab or nonspecific IgG to control for nonspecific Ab binding, and no 1° or 2° Ab to control for endogenous alkaline phosphatase contributing to the signal). We chose this two-day NGF treatment as a efficient and effective way to prepare our cells for robust functional assays of DAT level changes after E_2 _treatments.

### E_2 _inhibits DA uptake via the DAT

**Figure 2:**
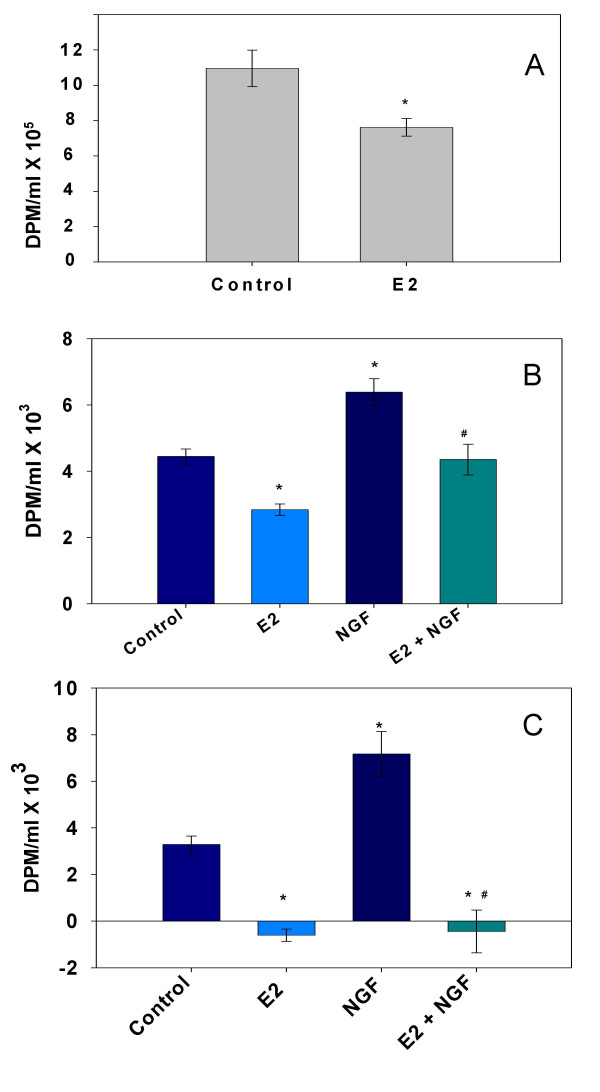
**DA uptake is inhibited by 10 nM E_2 _in PC12 cells**. Estradiol (E_2_), vs. ethanol vehicle (Control) was added to the cells together with ^3^H-DA for a 30 minute incubation, * = significantly different from vehicle control at p < 0.05. **A. **Cells were serum-starved for 48 hours with no NGFβ treatment. Total cellular uptake of DA over a 30 min period ± E_2 _treatment was monitored. **B. **Cells were serum-starved while being treated for 2 days ± 20 ng/ml NGFβ. DAT-specific DA uptake was measured over a 30 min period ± E_2 _treatment. NET- and DAT-blocking drugs were included to evaluate DAT-specific uptake of DA. # = significantly different vs. NGFβ-treated control (p < 0.05). **C. **The rapid effects of E_2 _treatment on ^3^H-DA uptake were shown when E_2 _treatment was added only during the last 5 minutes of the 30 min uptake assay. # = significantly different vs. NGFβ-treated vehicle control (p < 0.05).

We next addressed the affect of 10 nM E_2 _on DA uptake. First we looked at total DA uptake in PC12 cells (not blocked by any specific inhibitors to define a particular mechanism), and found that general uptake was inhibited by E_2 _(Fig 2A). We next added the specific DAT inhibitor nomifensine to define uptake specifically mediated by DAT (Fig. 2B). Again, E_2 _significantly blocked DAT-specific DA uptake. DAT-specific uptake was enhanced in NGF-treated cells, in agreement with the increased levels of DAT shown in Fig. 1. NGF-enhanced DAT activity was inhibited to a similar extent as the basal levels. Finally, though a 30-min treatment with E_2 _is considered a relatively short period of time for E_2 _to act (and so could be attributable to the nongenomic pathway), some may still argue that E_2_-induced transcription and translation could contribute to an effect in this time frame. Therefore, we next tested a shorter E_2 _exposure time during the 30-min uptake assay (Fig. 2C) to more clearly link this inhibitory function of E_2 _to a nongenomic mechanism of action. Again, treatment with NGF increased the DAT-specific uptake of DA, suggesting that new NGF-induced DAT is functional. When 10 nM E_2 _was added for only the last 5 min of the uptake assay, it dramatically inhibited DAT activity, more effectively than during the 30-min assay; uptake was completely reversed with only a 5-min hormone exposure. The NGF-inducible portion of the transport was also completely blocked by E_2 _treatment. Such effects not only show rapid and efficient E_2 _inhibition, but also suggest that the hormone may reverse the direction of the transporter, causing DA taken up in the previous 25 min to be removed from the cell.

### PC12 cells have message or protein of all three types of ERs located in membranes

**Figure 3:**
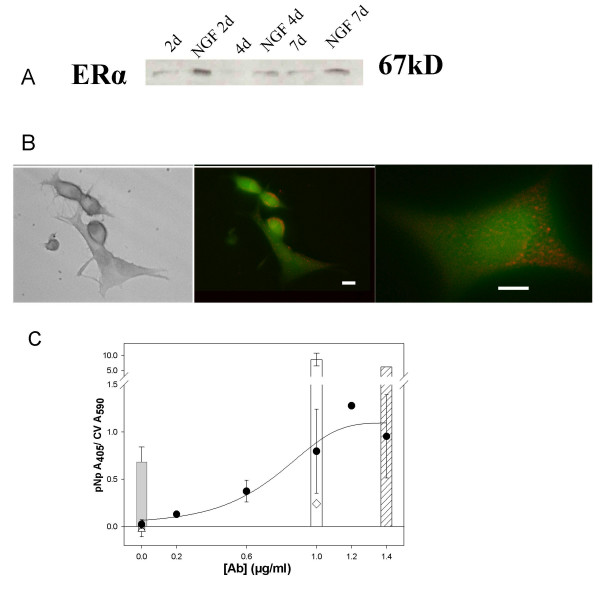
**PC12 cells have both membrane and intracellular ERα that is increased by NGFβ treatment**. ERα was detected with C542 Ab. **A. **Protein levels of ERα in whole-cell extracts were determined by immunoblot analysis at 2, 4 and 7 days ± NGFβ treatment in medium lacking serum. Representative of 3 experiments. **B. **Micrograph of immunocytochemical staining of ERα using nonpermeabilized cells that had been serum-starved for 48 hours while being treated with 20 ng/ml NGFβ. The bar represents 2 μm. Fluorescent images viewed with an FITC filter were photographed. Left panel: Transmission micrograph of middle panel. Middle panel: Immunocytochemistry of ERα shown in red (Vector Red product), while the autofluorescent background is green. Right panel: Punctate ERα staining is irregularly distributed on the cell surface of a more highly magnified cell. **C. **The fixed cell microplate immunoassay shows that ERα is present in PC12 cells grown in serum-containing medium. The values for nonpermeabilized cells are shown by symbols: ● different ERα Ab concentrations, △ combined control conditions (IgG isotype control, no 1°, and no 1°/no 2°), superimposed at the origin as their values are all very low and do not differ from each other significantly, ◇ clathrin. The values for permeabilized cells are shown by bars at the appropriate Ab concentrations. The crosshatched bar is ERα, the gray bar at the origin represents combined controls (see above), and the white bar is clathrin (the permeabilization indicator). This graph represents average values from 3 experiments ± S.E.M.

**Figure 4:**
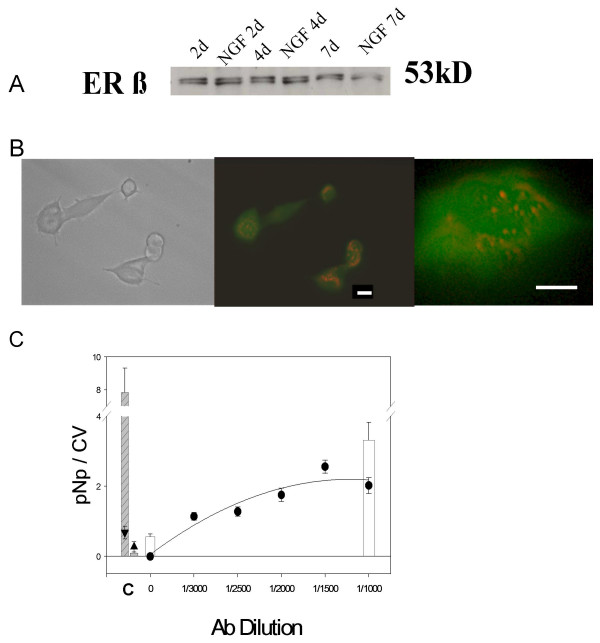
**PC12 cells have both intracellular and membrane ERβ**. Monoclonal Ab clone 9.88 was used for detection. **A. **Protein levels of ERβ in whole-cell extracts were determined by immunoblot analysis. ERβ is present in cells grown in serum-free medium, but is not induced by NGFβ over a 7-day period. Representative of 3 experiments. **B. **Immunocytochemistry of non-permeabilized cells treated with 20 ng/ml NGFβ for 2 days before staining. The bars represent 2 μm. Fluorescent image micrographs were viewed with an FITC filter were photographed. Left panel: Transmission image of middle panel. Middle panel: membrane ERβ immunofluorescence present heterogeneously on cells. Right panel: Punctate ERβ staining is irregularly distributed on the cell surface of a more highly magnified cell. **C. **Both nonpermeabilized cells (symbols) and permeabilized cells (bars) were assessed for ERβ by the plate assay over an Ab saturation curve. Controls (C) are as previously described in Figures 1 and 3. The cross-hatched bar is clathrin from permeabilized cells; ▼ = clathrin signal from unpermeabilized cells. The solid gray bar represents combined controls (IgGκ, no1°, no2° Abs) from permeabillzed cells and ▲ = the same combined controls in unpermeabilized cells. The white bars represent values for ERβ Ab 9.88 detection in permeabilized cells at the concentrations shown on the X axis. This graph represents average values from 2 experiments (each with multiple samples) ± S.E.M.

Next we wished to examine which specific subtypes of ERs may be present in PC12 cells and could be responsible for these rapid effects on DA transport. The presence of naturally expressed ERs α and β in PC12 cells and their long-term (weeks) upregulation by NGF have been previously reported [31,32,40,43], but not the presence of these proteins in the membrane. Our single protein band for ERα and the doublet bands seen for ERβ (Figs. 3a and 4a, Western blots) are similar to that seem by others [31] and confirm that ERα and ERβ are expressed in PC12 cells. The results in Fig. 3A also show that ERα can be elevated by a 2 day (or longer) NGF treatment. We were particularly interested in demonstrating membrane versions of ERs, as those are the most likely to participate in rapid nongenomic responses. Because these immunoblots contained ER protein from whole cell extracts, we next examined the subcellular location of these receptor proteins. As expected, ERα was shown to be in the nucleus of fixed, permeabilized cells (data not shown). ERα was heterogeneously present on membranes of fixed, nonpermeabilized cells (Fig. 3B), and appeared on both the cell body and processes, arranged in irregularly spaced punctate clusters. This is similar in appearance to the mERα staining that we have observed previously [44-46]. We next applied these nonpermeabilizing immunocytochemistry conditions to developing a quantitative plate assay that we have used previously to demonstrate mERα on other cells [35,47]. Fig. 3C shows saturability of the membrane antigen with increasing Ab concentrations, and comparisons of the low levels of membrane receptors to the levels of nuclear ERα measured with the same technique in cells permeabilized with detergent. The bars show the values for these proteins when cells are permeabilized, and the symbols within each bar show the same values in nonpermeabilized cells. As expected, the membrane population of these proteins is much smaller in each case than the whole-cell value in permeabilized cells. Negative controls (including no 1° Ab to detect any nonspecific binding of the 2° Ab, and no 1° or 2° Abs to detect any endogenous alkaline phosphatase contributing to the colorimetric signal) gave very low values. The clathrin signal in unpermeabilized cells was very low, and the permeabilized cell value was quite high, as expected for a protein residing just inside the plasma membrane.

The other classical ER family member, ERβ was then similarly investigated. In contrast to ERα, NGF treatment spanning 7 days did not affect the levels of this receptor protein (Fig. 4A). In Fig. 4B mERβ was assessed by immunocytochemistry on NGF-differentiated cells prepared with a nonpermeabilizing fixation technique. The appearance of mERβ was very similar to that of mERα – heterogeneous membrane staining among cells, punctate, and unevenly distributed over both cell bodies and processes. Again, measurement of this receptor was amenable to a quantitative plate assay (Fig. 4C) which showed a saturable receptor protein antigen in the membrane, with somewhat higher levels present in the whole (permeabilized) cells; low negative control values and high positive control values were similar to those shown for the ERα plate assay.

**Figure 5:**
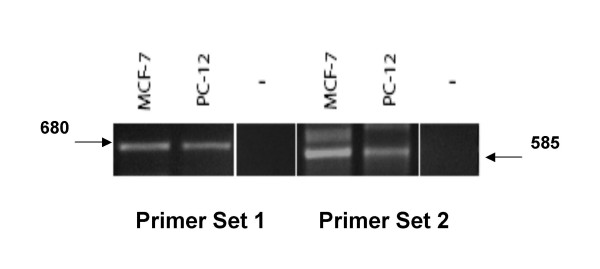
**GPR30 RNA is present in PC12 cells**. The presence of GPR30 ER RNA was detected by RT-PCR using two different primer sets compared to the human breast cancer cell line MCF-7, previously shown to express this receptor. Markers (1 kb DNA Ladder, Invitrogen) were used to determine that the amplimers were of the correct size of 680 and 585 bp, for primer set 1 and 2, respectively.

Finally, we examined RNA from these cells to determine if the rat GPR30 RNA was expressed; the lack of a specific Ab for rat GPR30 necessitated this approach. An RT-PCR amplimer of the anticipated size can be produced from PC12 cell RNA (Fig. 5) using two different sets of primers. This result matches those for the positive control cell line, MCF-7 human breast cancer cells. No signal was evident when RNA samples were omitted, showing that contaminated reagents did not produce this signal. Though this RT-PCR detection is not a quantitative method, the levels of GPR30 RNA in PC12 cells appear to be similar to that in MCF-7 cells.

### DA uptake is regulated by E_2 _dose, time of exposure to E_2_, and density of E_2_-exposed cells

**Figure 6:**
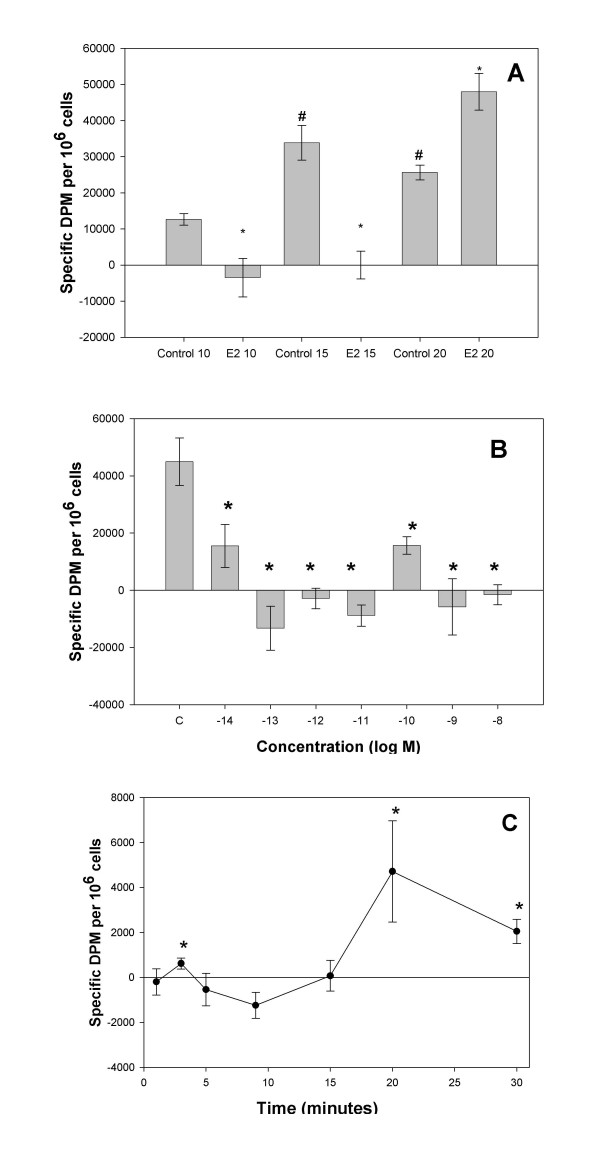
**Inhibition of DA uptake by E_2 _is regulated by cell density, and dose and time of E_2 _treatment**. Cells were serum-starved while being treated for 2 days with 20 ng/ml NGFβ. NET- and DAT-blocking drugs were included to define DAT-specific uptake. **A. **DA uptake inhibition by 10 nM E_2 _is robust at 10,000 and 15,000 cells/well, but reversed at densities as high as 20,000 cells/well. DAT-specific DA uptake was measured over a 30 min period ± E_2 _treatment during the last 5 minutes of the assay. * = significant difference between control and E_2_-treated samples at the level of p < 0.05. # = significant difference in DA uptake due to cell density conditions. **B. **All doses of E_2 _from 10^-14 ^to 10^-8 ^M caused inhibition of DA uptake, though with different levels of effectiveness, and in a nonconventional dose-response pattern. * = significant difference between control and E_2_-treated samples at the level of p < 0.05. **C. **The oscillating effects of 10 nM E_2 _on DA uptake at room temperature. Ethanol control background was subtracted from these values.

In previous studies we noted that mERα and the downstream effects it mediates in pituitary (GH3/B6/F10) cells were profoundly influenced by the density at which cells were grown; with increasing cell density, the expression of mERα (in favor of intracellular ERα) declined dramatically. Cells grown at higher densities (though not unusual densities for most cell culture experiments) also became unresponsive to E_2 _for nongenomic actions [46]. Therefore, we examined the effect of cells grown at various densities on the 5 min, 10 nM E_2_-induced inhibition of DA uptake in PC12 cells (Fig. 6A). Increasing the density from 10,000 to 15,000 cells per well of a 48-well plate increased the measurable DA uptake; E_2 _was still completely effective in inhibiting this higher level of uptake. However, when cells were further crowded to 20,000 cells per well, the inhibitory effect of E_2 _was lost, and instead an estrogenic stimulatory effect on DA transport was observed.

Because we have repeatedly observed non-conventional dose-response relationships for these nongenomic estrogenic responses, we always test our responses over a very wide (fM to nM) concentration range. Fig. 6B shows such an analysis for E_2_'s effects on the DA uptake response. As we have observed for rapid responses in other tissues [29,41,48,49], there is more than one peak of inhibitory activity for E_2 _in PC12 cells, separated by concentrations at which E_2 _is less effective (10^-10 ^M).

To determine the rapidity and stability of this estrogenic effect on DA transport, we examined a time course of 10 nM E_2 _exposure, concentrating on the more rapid (<30 min) increments expected to operate via the nongenomic mechanism. As we have seen many times in the past for nongenomic responses [41,47], rapidly oscillating temporal phases of this response were evident. Because these effects changed rapidly over time (from inhibition to enhancement of uptake) at 37°C, the results had somewhat large error ranges, as expected for such a fluctuating system over short time intervals when samples have to be removed from the incubator for analysis. Therefore, we reduced the temperature of the assay (to ambient) to hopefully create a more stable experimental demonstration of this oscillation. Under these conditions (Fig. 6C), peak inhibitory effects were seen at the 9 min time point. A small transport enhancement occurred at 3 min and a more robust one at 20–30 min.

### E_2 _affects DAT protein localization, but not overall cellular levels

**Figure 7:**
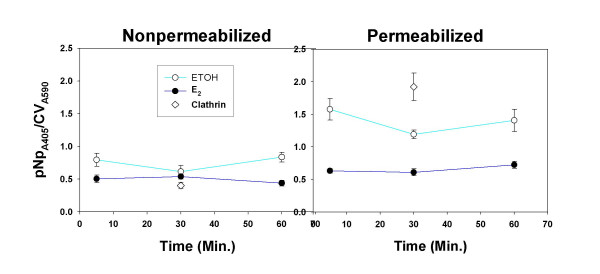
**Effects of acute E**_2_**treatment on DAT protein levels in the membrane vs. intracellular compartment using the fixed cell immunoplate assay**. After the cells had been serum-starved and NGFβ-treated for 2 days, 10 nM E_2 _or ethanol vehicle (ETOH) was added for the times indicated, before cell fixation and assay. Nonpermeabilizing conditions of fixation are shown in the left panel and permeabilizing conditions are shown in the right panel. ◇ represents clathrin Ab signal for monitoring the cell permeabilization status, as in previous figures. All E_2_-treated samples had DAT values significantly lower than ethanol-treated controls (p < 0.05).

**Figure 8:**
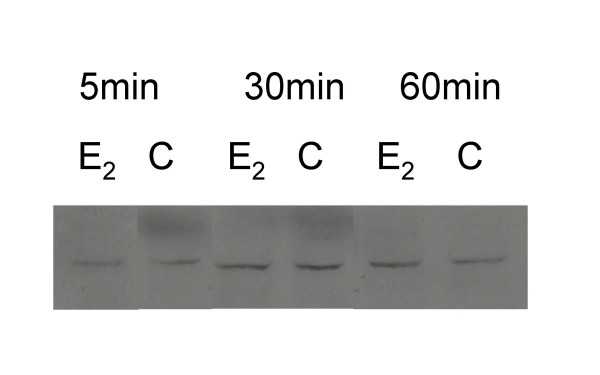
**DAT levels in whole-cell extracts are not affected by E_2 _treatment for 5–60 min**. Cells were serum-starved and NGFβ-treated for 2 days, then treated with 1 nM E_2 _vs. ethanol vehicle (C). Cell lysates were processed for immunoblot analysis with DAT Ab e2. DAT protein levels did not change due to E_2 _treatment over a 5–60 min time course. Representative of 2 experiments.

Because DAT-mediated uptake was inhibited by E_2_, we next examined whether this could be due to E_2_-induced DAT trafficking or reduced protein levels. We adapted our quantitative plate assay for DAT to measure cell surface vs. intracellular DAT, by permeabilizing the cells for the latter measurement with detergent during the cell fixation step. An Ab to the second extracellular loop of DAT recognizes transporter which is either on the plasma membrane or inside the cell, but not vesicle-bound (in which case the 2^nd ^loop would be facing the inaccessible vesicle interior). Fig. 7 shows that the amount of DAT was decreased in both of these cellular compartments. Next we looked at the whole-cell levels of DAT protein, to determine if E_2 _might have an effect on rapid DAT protein stability. Such rapid turnover sometimes occurs for phosphoproteins that after activation are subsequently ubiquitinated and sent to proteosomes or lysosomes. Fig. 8 shows that E_2 _treatment had no effect on DAT protein levels, compared to vehicle-treated cells, across a time course of 5 min to 1 hr.

## Discussion

Our studies demonstrated the expression of DAT and several types of membrane ERs (ERα, ERβ and GPR30) in PC12 cells. We demonstrated early (2 day) low-dose NGF enhancement of DAT and ERα levels, with functional consequences. Our qualitative and novel quantitative demonstration of membrane versions of ERα and ERβ in these cells suggests that they could be mechanistic mediators for the rapid E_2_-induced functional effects we saw; these effects are too rapid to be mediated by nuclear receptors via transcriptional controls. Furthermore, detection of GPR30 RNA expression in PC12 cells suggests that this newly described unique ER could also participate in these responses. We demonstrated that physiological levels of E_2 _(fM to 10 nM) can cause very rapid and dramatic inhibition of DA transport in PC12 cells. This is similar to rapid effects on serotonin transport that we previously described in RN46A cells [48]. These changes in DAT activity are not due to any major effects on turnover of the DAT protein, but instead probably operate via trafficking of DAT [50] into vesicles that protect its extracellular domains from Ab detection, or posttranslational modification mechanisms that remain to be investigated.

Can estrogen treatments alleviate human diseases which may involve DA transmission? Treatment with certain estrogens has been shown to relieve some cases of postpartum depression (reviewed [51]). However, the reasons for the failures in other cases, or a rationale for specific dose and estrogen choice is still not clear. Direct evidence supporting an estrogen treatment strategy includes simulation of pre- and post-partum estrogen levels [52]. There is a rat model for E_2 _reversal of depression [53], yet E_2 _therapy in humans is not always effective in reversing mood depression [52,54-56]. One explanation for these discrepancies could be that other prominent estrogen metabolites (estrone, estriol) are also involved in this function, and must be included as part of treatments to address mood maladies brought on by hormonal increases or deficits. In addition, the efficacy of such treatments could depend upon appropriate estrogen concentrations, and our studies have demonstrated once again [29,41,48,49] that a nonconventional dose-response relationship makes predictions of effective doses more difficult.

One of the regulatory effects we observed for this estrogen/DA signaling model system was the change in estrogenic response due to the extent to which cells were in contact with each other (brought on by increasing cell density). We have previously observed similar changes in effects due to increasing cell density – inhibition of mERα expression and linked responses in other cell types [46,47]. Experiments using cells grown at these higher inhibitory densities is quite common in most laboratories, and may be a common cause for negative reports for nongenomic signaling mechanisms and membrane steroid receptor detection. In addition, we observed these effects to oscillate over time, similar to our previous observations of other nongenomic effects. The mechanisms likely to be involved in such regulation (such as post-translational modifications of proteins) are also capable of rapid reversal and compensatory changes over time and in response to changes in cell-cell contact, such as those that occur during development. Clearly, much more information about the effects of such regulatory constraints on the nongenomic estrogenic response system and its receptor(s) are needed before we can take advantage of these mechanisms in therapeutic design. These data also demonstrate why precise dose-response and temporal response information is very important for determining safe and effective replacement therapies with estrogens. Because estrogens can increase the risk of cancers such as those of the breast, uterus [57], and pituitary [58], and may cause a decline in specific cognitive functions [59,60], dose and scheduling of replacements may be quite important. Though the protective effects of some estrogens against ischemic and glucocorticoid-induced brain injury have been demonstrated [61,62], such studies have focused on very high doses of estrogens that are unacceptable for chronic use because of the cancer risk. Therefore, we clearly do not yet understand the specifics of how estrogens act in the nongenomic pathway regarding tissue-specificity and non-classical dose-response patterns [63]. Thus, knowing parameters such as the lowest effective estrogen dose ranges and the kinetics of these response mechanisms are critical.

Our data suggest that one mechanism that may mediate E_2_-induced DA uptake inhibition is trafficking of DAT to an Ab-inaccessible site inside the cell. Recent reports show that DAT can be regulated by endocytosis [50,64-67]. In addition, agents that cause phosphorylation of DAT may regulate their trafficking by sequestration to particular intracellular compartments [50,67,68]. PKC probably mediates these effects via modification of a C-terminal pentapeptide sequence with homologies between DAT, SERT and the norepinephrine (NET) transporters [50]. E_2 _is known to activate protein phosphorylating enzymes (including PKC) via nongenomic pathways (reviewed in [69]), and so could affect the functions of DAT (and other transporters of this family) via this mechanism. Phosphorylation also sometimes marks proteins for ubiquitin-tagging, removal to proteosomes, and degradation [67,70]. This does not appear to be the case in our experimental system, as no noticeable DAT protein loss occurred over short-term E_2 _exposure. However, it should be noted that immunoblot quantitation seemed to be the least sensitive of our assays in detecting changes in protein levels. But since no dramatic changes in levels were evident, it is more likely that the direct estrogenic effects in our system are on activity and localization of the DAT machinery.

Our observations of candidate ER proteins in the plasma membrane of PC12 cells, coincident with a rapidly mediated estrogenic effect, adds yet another example to our growing list of such steroid-regulated systems employing the nongenomic pathway of action. We have done extensive studies on the cellular localization of the ERα in the GH_3_/B6/F10 cell model and visualized the membrane version of this receptor by a variety of techniques [34,35,44,45]. We have also recently extended these studies to ERα in MCF-7 cells [71] and we developed a similar story on the expression of the membrane glucocorticoid receptor [72,73] in lymphoid tissues. Overall, we find that the relative number (fewer membrane receptors compared to nuclear receptors), distribution, and appearance of these apparently clustered groups of receptors is similar, and agrees with membrane steroid receptor characterizations by other investigative groups (for example, [74,75]). However, the presence of mERβ in PC12 cells is unique among cells we have studied using these techniques; mERβ has only previously been observed in cells over-expressing an ERβ cDNA [36]. Our observation of the presence of GPR30 RNA in these cells presents a third possibility for regulation of DAT by an ER perhaps co-resident in the plasma membrane of these cells [76]. Future studies will examine the specific roles of each of these membrane receptor subtypes in the regulation of the DAT.

## Conclusion

In summary, elucidating the underlying cellular mechanisms and receptors that are responsible for steroid regulation of neurotransmitter transporter functions will be critical for medical decision-making about the appropriate amount and type of hormones administered for therapeutic benefit. This will be critically important for such common conditions as post-surgical or post-menopausal estrogen loss, and monthly or pregnancy cycle fluctuations. We must understand the basic actions of physiological estrogens such as E_2 _on this system, so that analogs or antagonists can be utilized to alleviate life-stage-specific estrogenic effects or deficits. A new focus on nongenomic steroid effects may allow entirely new approaches to treating these maladies, and explain which physiological estrogens at what doses should be considered in the diagnosis and treatment of these diseases.

## Methods

### Cell culture and hormone administration

We propagate our PC12 cells in 15% serum-containing medium, releasing them for subculture by repeatedly pipetting the cells. However, before each experiment, cells were transferred to a defined medium for 48 hrs to insure withdrawal from the effects of estrogens and other hormones and growth factors present in serum. Our defined medium is high-glucose, phenol red-free RPMI 1640 (Gibco/Invitrogen, Grand Island, NY), substituting the 15% serum with TCM serum replacement (Celox Laboratories, St. Paul, MN). During this serum starvation period NGFβ (a gift from Dr. Regino Perez-Polo) was added to (at 20 ng/ml) to designated cultures for these studies. We study "native" cell regulation using NGF stimulation to achieve levels of DAT in our cells that can be easily assayed for the inhibitory effects of E_2_, instead of transfecting cells with constructs that will over-express DAT. In this way our studies differ from many previously published DAT regulation studies. Under these conditions, regulatory interactions may be subject to fewer artifacts due to over-expression, such as normal receptor level interactions with other signaling molecules.

### Immunocytochemistry

PC12 cells plated on poly-D-lysine (10 μg/ml)-coated coverslips were cultured in 6-well plates at densities of 20,000–40,000 cells per well for 24 hours. After serum starvation ± NGF treatment, cells were fixed using 2% paraformaldehyde (Fisher Scientific, Houston, TX), and 0.2% gluteraldehyde (Electron Microscopy, Fort Washington, PA) for 30 minutes. Where applicable, cells were permeabilized during fixation by adding NP-40 (or IGEPAL CA-630 can be substituted) and sucrose [35]. Unquenched aldehydes were reduced by applying a 13 mM NaBH_4_, 70 mM NaHPO_4 _aqueous solution for 15 minutes at room temperature. For additional reduction of autofluorescence background, Schiff's Reagent (Fisher) was applied for 15 minutes while on ice, and then rinsed off three times with sulfurous water (equal volumes of 1 N-HCl and 10% sodium metabisulfite aqueous solution). The wells were washed (for 10 minutes) with a second reducing solution (1%Na_2_HPO_4_/1%NaBH_4 _in ddH_2_O) at room temperature, with light shaking. This was followed by a 15 minute PBS wash. Cells were then blocked for 45 minutes at RT with 0.1% coldwater fish gelatin (Sigma, St. Louis MO) in PBS. The cells were incubated overnight at 4°C (with light orbital shaking) with primary Ab (diluted in fish gelatin/PBS); the 1° Ab we used for detection of ERα, at concentrations of 2–10 μg/ml was C542 (StressGen, Inc., Collegeville, PA). Anti-ERβ monoclonal Ab (clone 9.88, Sigma E1276) was used at dilutions ranging from 1:500–1:2000. Ab DAT/e2 to the DA transporter binds to the second extracellular domain [42], and was obtained from A. Levey (Emory Univ.). It was used at concentrations ranging from 0.025–10 μg/ml. Anti-clathrin Ab (ICN Biochemicals, Inc., Aurora, OH) was used in concentrations of 1–3 μg/ml as a control for cell permeabilization. We used mouse IgG1κ (Sigma) as an irrelevant Ab and isotype control. Secondary Ab (either biotinylated mouse IgG or IgM, from Vector Laboratories, Burlingame, CA) was diluted (50 μl/10 ml) in 0.1% fish gelatin/PBS and incubated with samples for 1 hour at RT. Using a kit from Vector Laboratories, ABC-AP solution (diluted in PBS) was added, followed by a series of PBS washes, and then Vector Red alkaline phosphatase substrate (Vector Laboratories) was added for 2–5 minutes. The alkaline phosphatase substrate was prepared by adding 2 drops of each reagent from the kit to 5 ml of 100 mM Tris-HCl (pH 8.2–8.5) and adding the endogenous phosphatase inhibitor levamisole (Vector Labs) to a final concentration of 0.5 mM. The solution was removed and the wells immediately rinsed with ddH_2_O; a small volume was left in each well until the coverslip was ready to go through the following cycle: dehydration with two changes of 70% ethanol (30 seconds each), followed by two changes of 100% ethanol (30 seconds each). The coverslips were then cleared with three xylene washes (3 minutes each) and then mounted with Cytoseal 280 (Electron Microscopy). Images were viewed under an FITC filter with a Leitz fluorescence microscope equipped with a CoolSNAP-Pro digital camera from Media Cybernetics using Image-Pro Plus software. The cell images were viewed using a wide-spectrum FITC filter. Under these conditions the intense red staining of Vector Red is observed as orange red staining; the green staining is aldehyde-induced autofluorescence background. We used these photographs with included autofluorescence background to give a simultaneous cell outline upon which the Ab-mediated Vector Red signal is visible. Specific staining was also visible using a rhodamine filter, but without the green background (not shown).

### Western blotting

PC12 cells were grown to 50% confluence in 100 mm Petri dishes and serum-starved ± NGF for 2–7 days. Cells were then rinsed twice with ice-cold PBS and solubilized in 0.5 ml of lysis buffer (20 mM Tris; 150 mM NaCl; 1 mM EDTA; 1 mM EGTA; 1% Triton X-100; 2.5 mM sodium pyrophosphate; 1 mM β-glycerolphosphate; 1 mM Na_3_VO_4_; 1 μg/ml leupeptin; 1 mM PMSF) at 4°C. After sonication (4 × s, 5 sec each), the insoluble materials were removed by centrifugation at 15000 × g for 10 min. The extract was treated with SDS sample buffer and boiled for 5 min. Aliquots were assayed for protein concentration (BioRad), and 20 μg/ml total protein was subjected to SDS-PAGE in 10% acrylamide, and then transferred to a nitrocellulose membrane. Blots were blocked (2% nonfat dry milk, 1% BSA in 10 mM Tris-buffered saline pH 7.4) for 1 hour, followed by overnight incubation with primary Ab for ERα (1 μg/ml Stressgen, SRA 1010), ERβ (1:1000, Sigma E1276), or DAT (DAT/e2, 1:500 from A. Levey) at 4°C. Blots were then rinsed and incubated with peroxidase-conjugated anti-mouse IgG (1:4000, Southern Biotech) for ERα and ERβ, and peroxidase-conjugated anti-rat (1:4000, Southern Biotech) for DAT at RT for 2 hours. Immunoreactivity was detected on X-ray film (Amersham) by enhanced chemiluminescence.

### Plate immunoassay for detection/quantitation of DAT and Ers

We originally developed sensitive and specific quantitative fixed cell sandwich immunoassays suitable for 96-well plates for demonstrating mERα on the cell surface of both GH3 cells and MCF-7 breast cancer cells [35,47]. For the present studies we further adapted the assay for use with PC12 cells, to measure DAT, ERa and ERβ. Cells were plated in serum-containing medium, fed with serum-free defined medium for 48 hrs, then fixed with 2% paraformaldehyde/0.1% glutaraldehyde. Cells were then treated with the following reagents, with washing steps between each application: NaBH_4 _for free aldehyde reduction; fish gelatin for blocking; 1° Ab; biotinylated 2° Ab; avidin-conjugated alkaline phosphatase; and levamisole (to block the endogenous mammalian subtype of alkaline phosphatase). Then pNpp, a substrate for alkaline phosphatase, was added, producing a soluble yellow dephosphorylated product (pNp) measured at A_405 nm_. Finally, the pNp reagents were washed from the wells, and the cells stained with crystal violet (CV); after extraction, this dye was read at A_590 nm _as a measure of cell number, to which our antigen values were normalized.

This assay was adapted to measure the same antigens in the intracellular compartment by simply including membrane-permeabilizing detergents in the fixation step, so that levels of intracellular (permeabilized) and extracellular (unpermeabilized) antigens can be compared by essentially the same methods [35]. We thus assayed membrane antigens for intracellular relocation (trafficking). Ab for the abundant intracellular antigen clathrin was used to determine the permeabilization status of the cells in each assay.

### Measurement of DA transport [77]

Cultured PC12 cells (1–4 × 10^4^/well in poly-D-lysine-coated 48-well plates) were rinsed and preincubated in buffer for 30 min under assay conditions at 37°C. Inhibitors were included in this preincubation step. Transport assays were initiated by adding buffer containing ^3^H-DA (Dupont NEN, 50 nM for single point assays), monoamineoxidase inhibitors pargyline or selegeline [78], ascorbic acid (for metabolic stability of E_2 _and other compounds), and 50 nM DMI (to inhibit any contribution from the NET). The specific DAT inhibitors nomifensine (500 nM) or GBR-12909 (100 nM) were included in parallel samples; the differences between responses ± these inhibitors were used to define uptake due to DAT. In some cases E_2 _was added concomitantly with the labeled DA; in other cases it was added after the labeled DA incubation had continued for number of minutes; thus E_2 _was added only for the last min of the uptake assay. Assays were terminated by rapidly washing the wells 3 × with ice-cold buffer. Cells were then solubilized in water for 15 min at room temperature with shaking, or by a freeze-thaw cycle, and an aliquot assessed for ^3^H via liquid scintillation counting. In some cases, another aliquot was assayed for protein content with a Bio-Rad Bradford assay; this value determined wells where cells had become disengaged from the well bottom, for data exclusion. Uptake assays to determine the temporal changes in regulation were done at 15,000 cells per well and at room temperature, slowing the reaction and allowing for less error prone measurements.

### RNA isolation and PCR analysis

RNA was prepared from PC12 cell lysate using the RNAqueous kit (Ambion). First-strand cDNA synthesis was performed using the SuperScript III First Strand Synthesis System for RT-PCR (Invitrogen). Briefly, 2 μg RNA, 50 μM oligo(dT), 10 mM dNTP mix, and DEPC-treated water up to 10 μl final volume was incubated at 65°C for 5 minutes and placed on ice for 1 minute. The samples were then incubated for 50 minutes at 50°C with the addition of 10 μl of cDNA synthesis mix containing RT buffer, 25 mM MgCl_2_, 0.1 M DTT, RNaseOUT, and Superscript III RT (200 U/μl). The reaction was terminated by heating the samples to 85°C for 5 minutes. The PCR reaction was performed in 25 μl GoTaq Flexi DNA Polymerase buffer (Promega) containing 0.2 μM of both gene specific sense and antisense primers, plus 0.5 μl of the RT reaction. The primers (kind gift of Dr. Peter Thomas [39]) were designed using GenBank sequence accession no. BC011634. Primer set 1 was: sense, 5'-GGC TTT GTG GGC AAC ATC-3'; antisense, 5'-CGG AAA GAC TGC TTG CAG G-3'. Primer set 2 was: sense, 5'-GCA GCG TCT TCT TCC TCA CC-3'; antisense, 5'-ACA GCC TGA GCT TGT CCC TG-3'. The PCR product was obtained using the GeneAmp PCR system 9700 with 35 cycles of 30 sec at 94°C, 30 sec at 55°C, and 2 min at 72°C followed by a 10-min extension at 72°C. The PCR products were electrophoresed in a 1% agarose gel containing 0.5% ethidium bromide, and bands corresponding to the anticipated products of 680 bp (primer set 1) and 585 bp (primer set 2) were identified.

### Statistics

A one way ANOVA (SigmaStat 3.0) was used to determine the significance of treatment effects compared to vehicle controls. Statistical significance was accepted at the p < 0.05 level.

## Competing interests

CW is a member of the scientific advisory board for CertiChem Inc.

## Authors' contributions

BH, AJ, and TR carried out the protein identification and quantitation studies. BH and RA performed the DA uptake studies. MT and KC helped in the development of these studies, provided financial support, and frequently assisted in experiment design and discussion of results. CW was the project leader and designer of this study, and participated in all aspects. All authors read and approved the final manuscript.
